# The fusion of vegetation indices increases the accuracy of cotton leaf area prediction

**DOI:** 10.3389/fpls.2024.1357193

**Published:** 2024-07-04

**Authors:** Xianglong Fan, Pan Gao, Mengli Zhang, Hao Cang, Lifu Zhang, Ze Zhang, Jin Wang, Xin Lv, Qiang Zhang, Lulu Ma

**Affiliations:** ^1^ Key Laboratory of Oasis Ecological Agriculture of Xinjiang Production and Construction Corps, Agricultural College, Shihezi University, Shihezi, Xinjiang, China; ^2^ College of Information Science and Technology, Shihezi University, Shihezi, Xinjiang, China; ^3^ Aerospace Information Research Institute, Chinese Academy of Sciences, Beijing, China

**Keywords:** canopy spectrum, fusion of vegetation indices, machine learning, monitoring, LAI

## Abstract

**Introduction:**

Rapid and accurate estimation of leaf area index (LAI) is of great significance for the precision agriculture because LAI is an important parameter to evaluate crop canopy structure and growth status.

**Methods:**

In this study, 20 vegetation indices were constructed by using cotton canopy spectra. Then, cotton LAI estimation models were constructed based on multiple machine learning (ML) methods extreme learning machine (ELM), random forest (RF), back propagation (BP), multivariable linear regression (MLR), support vector machine (SVM)], and the optimal modeling strategy (RF) was selected. Finally, the vegetation indices with a high correlation with LAI were fused to construct the VI-fusion RF model, to explore the potential of multi-vegetation index fusion in the estimation of cotton LAI.

**Results:**

The RF model had the highest estimation accuracy among the LAI estimation models, and the estimation accuracy of models constructed by fusing multiple VIs was higher than that of models constructed based on single VIs. Among the multi-VI fusion models, the RF model constructed based on the fusion of seven vegetation indices (MNDSI, SRI, GRVI, REP, CIred-edge, MSR, and NVI) had the highest estimation accuracy, with coefficient of determination (R2), rootmean square error (RMSE), normalized rootmean square error (NRMSE), and mean absolute error (MAE) of 0.90, 0.50, 0.14, and 0.26, respectively.

**Discussion:**

Appropriate fusion of vegetation indices can include more spectral features in modeling and significantly improve the cotton LAI estimation accuracy. This study will provide a technical reference for improving the cotton LAI estimation accuracy, and the proposed method has great potential for crop growth monitoring applications.

## 1 Introduction

Leaf area index (LAI) is a prominent influencing factor of vegetation photosynthesis and carbon cycling (Fang et al., 2019). It has been used as the most important indicator for canopy photosynthesis and energy exchange (Parker, 2020). However, accurate prediction of crop LAI remains a challenge in crop growth monitoring, production management, and yield estimation. Traditionally, LAI is measured by destructive sampling. This method is costly, labor intensive, time consuming, error-prone, and susceptible to human factors. In recent years, with the rapid development of remote sensing technology, hyperspectral remote sensing can be used to acquire detailed and rich spectral information, which can be used to quantitatively analyze the weak spectral differences of features. It has proved to have strong advantages in crop species identification, crop growth parameter inversion, yield estimation, and pest and disease monitoring (Zhu et al., 2020; Liu et al., 2021). Therefore, in this study, hyperspectral remote sensing was used to predict LAI changes.

Vegetation index (VI) is constructed by combining spectral features. In LAI prediction, the correlation between LAI and plant canopy spectral reflectance is determined first, then spectral features are screened out to construct VI. Finally, the linear or nonlinear relationship is used to estimate LAI (Li et al., 2018). Because VI can obviously reduce noises, at present, VI is widely used for the inversion of plant LAI (Qiao et al., 2020). For example, Ma et al. (2022) estimated cotton LAI by constructing NDVI, RVI, and DVI and found that DVI had the highest correlation with LAI (correlation coefficient, −0.76). Nie et al. (2023) assessed the stability and applicability of different VIs in LAI prediction for different crops and discovered that SRI (simple ratio index) had the highest accuracy in estimating cotton and winter wheat LAI, and MTCI (MERIS terrestrial chlorophyll index) had the highest accuracy in estimating maize LAI. Based on the same modeling process and parameters, Dong et al. (2019) found that the VI constructed based on red-edge reflectance had good performance in estimating the LAI of different crops. It can be seen that the correlation between LAI and VI and the modeling results for different crops are different.

However, the accuracy of a single VI in predicting LAI is not high (Yang et al., 2017). For example, Chemura et al. (2018) estimated the nitrogen content of coffee leaves by Sentinel-2 MSI spectral data and found that the estimation accuracy of the model constructed by RNDVI (R^2^, 0.48) was lower than that of the model directly constructed using spectral features (R^2^, 0.57). Xing et al. (2020) estimated the LAI by constructing triangular vegetation index (TTVI) and 14 conventional vegetation indices and found that although the accuracy of the model constructed by TTVI was higher than that of the models constructed by the conventional VIs, the accuracy was still low (the highest R^2^ was only 0.60). Wang et al. (2023) found that the chlorophyll spectral features of *Sabina vulgaris* Ant. in Mu Us Sandy Land had higher correlations with normalized vegetation index (NDVI), ratio vegetation index (RVI), and modified normalized vegetation index (mNDVI), and the difference between the accuracy of the constructed univariate linear regression model (R^2^ = 0.9) and the lowest accuracy (R^2^ = 0.1) was large, indicating a high instability of the model. The low accuracy and high instability of the models built by individual VIs may be due to the fact that a single VI contains few spectral features, and shortwave infrared (SWIR) spectra are susceptible to the influences of soil moisture, vegetation moisture, and atmospheric moisture (Thompson et al., 2018; Degerickx et al., 2019; Srinet et al., 2019), thereby reducing the accuracy and instability of spectral estimation. In addition, using all hyperspectral data for modeling can easily lead to information redundancy and model overfitting, which reduces the accuracy, versatility, and stability of the model (Moharram et al., 2023). It should be noted that multiple VI fusions could provide rich spectral information, and the constructed model could integrate the advantages of these VIs (Barzin et al., 2020); besides, the optimal VI fusion method could highlight the spectral features, significantly increasing the prediction accuracy. Therefore, it is necessary to explore the potential of multiple VI fusions in estimating cotton LAI, which is crucial to improve the accuracy of cotton LAI estimation and accurately monitor cotton growth.

In summary, although individual VIs have been widely used to estimate crop LAI, the estimation using an individual VI is easily affected by factors such as soil background and light conditions, which ultimately reduces model universality and stability. Especially, individual VIs contain limited spectral information, which always results in a low accuracy of VI estimation. In this study, a ML-based cotton LAI estimation model was constructed by fusing multiple VIs, and the optimal fusion model was selected. The specific objectives of the study were as follows: (1) to determine the VIs participating in the modeling through correlation analysis; (2) to select the optimal estimation model by constructing cotton LAI estimation models using machine learning modeling strategies (ELM, RF, BP, MLR, and SVM) based on the selected VIs; and (3) to randomly arrange and fuse the VIs participating in the modeling (i.e., for index 1, 2, and 3, there were seven fusions including 1, 2, 3, 1 + 2, 1 + 3, 2 + 3, and 1 + 2 + 3) to determine the optimal VI fusion method based on the selected optimal model. This study will provide a technical reference for large-scale rapid cotton growth monitoring and yield estimation.

## 2 Materials and methods

### 2.1 Study site and experimental design

**Figure 1:**
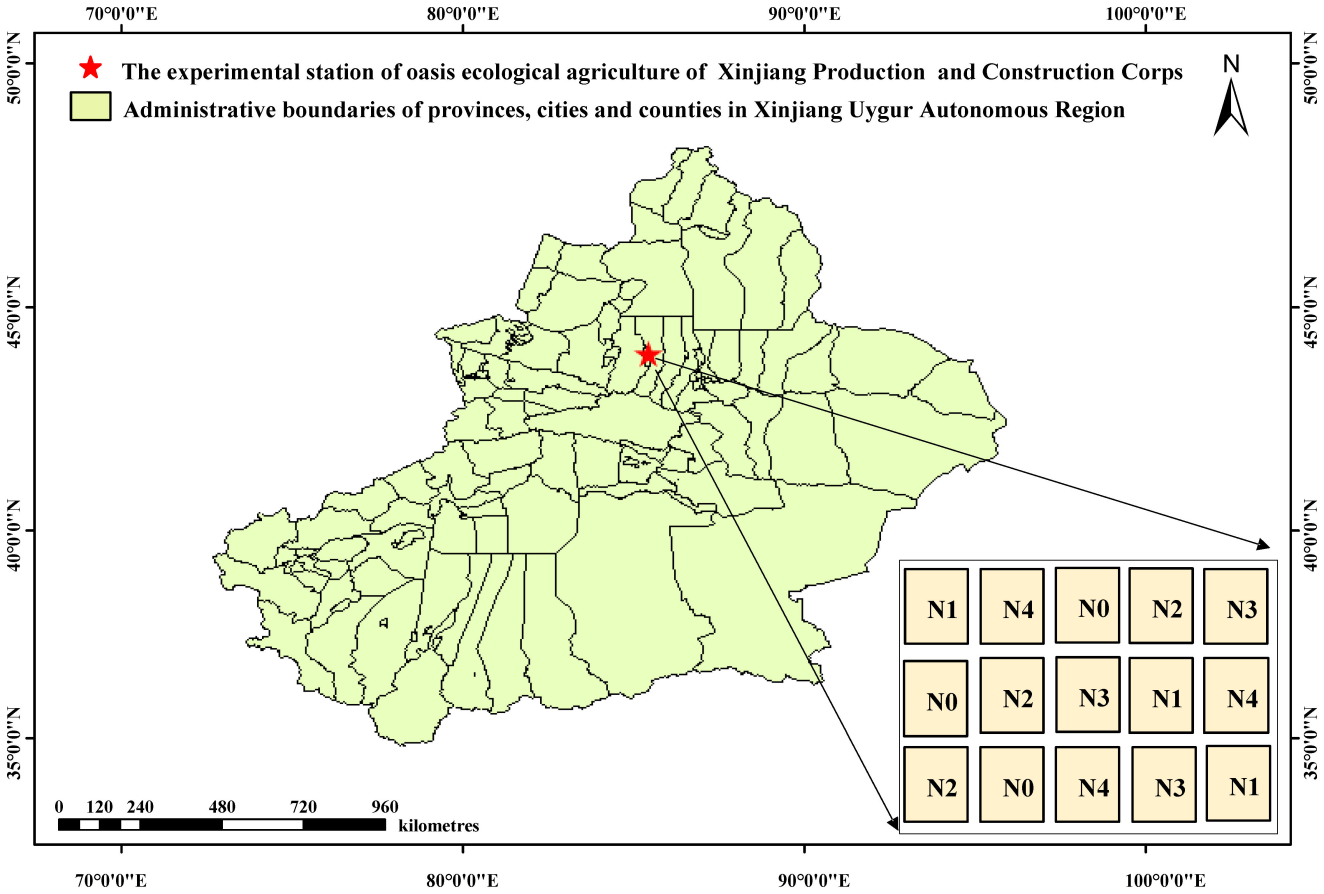
Location of test area and experimental design. N0, no nitrogen (N) fertilizer was applied; N1, N fertilizer application rate was 110 kg ha^−1^; N2, N fertilizer application rate was 220 kg ha^−1^; N3, N fertilizer application rate was 330 kg ha^−1^; N4, N fertilizer application rate was 440 kg ha^−1^.

The research was carried out in the Shihezi, China (86.03°E, 44.18°N, a.s.l. 429 m) (**Figure 1**). This region has a typical temperate continental climate. The average annual precipitation was only 125.9–207.7 mm, and the annual accumulated temperature (≥10°C) was 3,570°C–3,729°C. In addition, there was a large day–night temperature difference. The soil type was loam. Soil organic matter, alkaline hydrolyzable nitrogen, available phosphorus, and available potassium content were 20.10 g/kg, 60.92 mg/kg, 17.83 mg/kg, and 142 mg/kg, respectively. The previous crop was cotton.

In late April 2021, cotton seeds (variety Xinluzao 45) were sown, and drip irrigation and plastic film mulching were adopted [six rows and three irrigation tapes were under the mulching of one film (2.35 m in width)].

The randomized complete block design was adopted in this experiment, including five N treatments: 0 kg·ha^−1^ [N0 (CK)], 110 kg·ha^−1^ (N1), 220 kg·ha^−1^ (N2), 330 kg·ha^−1^ (N3, conventional application rate), and 440 kg·ha^−1^ (N4). Each treatment had three replicates (plots). Each plot was 70.5 m^2^ (7.05 m × 10 m), and the plot spacing was 4.7 m. Urea (N, 46%) of 345 kg·ha^−1^ and potassium dihydrogen phosphate (P_2_O_5_, 52%; K_2_O, 34%) of 240 kg·ha^−1^ were applied. The first fertilization was conducted on June 12, and fertilizers were applied every 8–9 days thereafter (nine times totally).

### 2.2 Acquisition of cotton canopy spectrum

The canopy spectrum of cotton plants without pests and diseases were collected at 12:00–14:00 on sunny days at the budding stage (June 21), flowering stage (July 10), initial bolling stage (July 25), and full bolling stage (August 18) using an ASD-FieldSpec Pro FRTM spectrometer. During acquisition, the probe of the sensor was downward, the vertical height was 50 cm, the sensor angle was 25°, and the scanning time was 0.2 s. Three sampling points were selected in each plot, and spectral acquisition was performed five times for each point. Finally, the average was calculated, which was the spectrum of the sample point. Spectral acquisition were conducted eight times totally during the whole growth period, and a total of 360 data were obtained. Previous studies have shown that the spectrum at 1,800 nm–2,500 nm is greatly affected by soil background and air moisture (Chandrasekar et al., 2022). Therefore, in this study, only spectral changes at 350 nm–1,800 nm were analyzed.

### 2.3 Determination of leaf area index 

After spectral acquisition, three cotton plants were selected from each plot. Then, leaves were separated, placed in a crisper box, and sent to the lab for LAI determination using a LI-3100 leaf area meter. Eight samplings were conducted during the whole growth period, and a total of 360 cotton plant samples were collected. The calculation method of LAI is shown in Equation 1:


1 $$\text{LAI}=\frac{\text{K}0\times \text{N}}{\text{K}}$$

where K0 is the representative of cotton leaf area in a plot, N is the cotton plant number of one plot, and K is plot area.

### 2.4 Model establishment

The VIs have been widely used to monitor vegetation information. Especially, VIs can distinguish non-vegetation information such as water bodies and enhance vegetation information ((Donovan et al., 2021; (Morsy et al., 2022). In this study, 20 VIs (**Table 1**) were selected for correlation analysis.

**Table 1:** Vegetation indices tested in this study.

Index	Equation	References
Normalized vegetation index (NDVI)	$\frac{{\text{R}}_{800\text{nm}}-{\text{R}}_{680\text{nm}}}{{\text{R}}_{800\text{nm}}{+\text{R}}_{680\text{nm}}}$	Tucker, 1979
Green Normalized Difference Vegetation Index (GNDVI)	$\frac{{\text{R}}_{800\text{nm}}-{\text{R}}_{550\text{nm}}}{{\text{R}}_{800\text{nm}}{+\text{R}}_{550\text{nm}}}$	Gitelson et al., 1996
Renormalized Difference Vegetation Index (RDVI)	$\frac{{\text{R}}_{800\text{nm}}-{\text{R}}_{680\text{nm}}}{\sqrt{{\text{R}}_{800\text{nm}}{+\text{R}}_{680\text{nm}}}}$	Roujean and Breon, 1995
Soil-Adjusted Vegetation Index (SAVI)	$(1+0.5)\times \frac{{(\text{R}}_{800\text{nm}}-{\text{R}}_{670\text{nm}})}{{(\text{R}}_{800\text{nm}}{+\text{R}}_{670\text{nm}}+0.5)}$	Haboudane et al., 2004
Modified Simple Ratio (MSR)	( $\frac{{\text{R}}_{800\text{nm}}}{{\text{R}}_{680\text{nm}}}-1$ )/ $\sqrt{\frac{{\text{R}}_{800\text{nm}}}{{\text{R}}_{680\text{nm}}}+1}$	Xie et al., 2016
Triangular Vegetation Index (TVI)	$0.5\times [120\times ({\text{R}}_{750\text{nm}}-{\text{R}}_{550\text{nm}})-200\times ({\text{R}}_{670\text{nm}}-{\text{R}}_{550\text{nm}})]$	Haboudane et al., 2004
Optimized Soil-Adjusted Vegetation Index (OSAVI)	$(1+0.16)\times \frac{{(\text{R}}_{800\text{nm}}-{\text{R}}_{705\text{nm}})}{{\text{R}}_{800\text{nm}}{+\text{R}}_{670\text{nm}}+0.16}$	Rondeaux et al.,1996
Modified Normalized Difference Vegetation Index (mNDVI)	( ${\text{R}}_{800\text{nm}}-{\text{R}}_{680\text{nm}}$ )/( ${\text{R}}_{800\text{nm}}{+\text{R}}_{680\text{nm}}-{2\times \text{R}}_{445\text{nm}})$	Huete et al.,1997
MNDSI (Modified Normalized Difference Spectral Index)	$\frac{\left({\text{R}}_{940\text{nm}}-{0.8\times \text{R}}_{950\text{nm}}\right)-{\text{R}}_{730\text{nm}}}{\left({\text{R}}_{940\text{nm}}-{0.8\times \text{R}}_{950\text{nm}}\right){+\text{R}}_{730\text{nm}}}$	Cao et al., 2017
NDI (Normalized Difference Index)	$\frac{{\text{R}}_{712\text{nm}}-{\text{R}}_{674\text{nm}}}{{\text{R}}_{712\text{nm}}{+\text{R}}_{674\text{nm}}}$	Delegido et al., 2013
RVI (Ratio Vegetation Index)	$\frac{{\text{R}}_{810\text{nm}}}{{\text{R}}_{560\text{nm}}}$	Aparicio et al., 2000
EVI (Enhanced Vegetation Index)	$2.5\times \frac{{(\text{R}}_{800\text{nm}}-{\text{R}}_{670\text{nm}})}{{\text{R}}_{800\text{nm}}{+6\times \text{R}}_{670\text{nm}}-{7.5\times \text{R}}_{475\text{nm}}+1}$	Zhang et al., 2020
DSI (Difference Spectral Index)	${\text{R}}_{760\text{nm}}-{\text{R}}_{739\text{nm}}$	Tanaka et al., 2015
SRI (Simple ratio indices)	$\frac{{\text{R}}_{750\text{nm}}}{{\text{R}}_{550\text{nm}}}$	Ranjan et al., 2012
GRVI (Green Ratio Vegetation Index)	$\frac{{\text{R}}_{800\text{nm}}}{{\text{R}}_{550\text{nm}}}$	Mao et al., 2020
NVI (New Vegetation Index)	$\frac{{\text{R}}_{777\text{nm}}-{\text{R}}_{747\text{nm}}}{{\text{R}}_{673\text{nm}}\;}$	Gupta et al., 2001
REP (Red edge position)	$700+\frac{40\times \text{R}\left(\frac{{\text{R}}_{670\text{nm}}+{\text{R}}_{780\text{nm}}}{2}-{\text{R}}_{700\text{nm}}\right)}{{(\text{R}}_{740\text{nm}}-{\text{R}}_{700\text{nm}})}$	Li et al., 2013
CI_red-edge_ (Chrolophyll Red-Edge Index)	$\frac{{\text{R}}_{800\text{nm}}}{{\text{R}}_{710\text{nm}}}-1$	Swain et al., 2013
MTVI (Modified Triangular Vegetation Index)	$1.2\times [1.2\times ({\text{R}}_{800\text{nm}}-{\text{R}}_{550\text{nm}})-200\times ({\text{R}}_{670\text{nm}}-{\text{R}}_{550\text{nm}})]$	Haboudane et al., 2004
RES (Red Edge Symmetry)	$\frac{{\text{R}}_{718\text{nm}}-{\text{R}}_{675\text{nm}}}{{\text{R}}_{755\text{nm}}{\;+\text{ R}}_{675\text{nm}}}$	Ju et al., 2010

### 2.5 Correlation analysis

To explore the interaction between VIs and the correlations between VIs and LAI, a correlation matrix between VIs and LAI was generated using Origin. Pearson correlation coefficient indicates the level of correlation (−1–1). The higher the absolute value, the closer the correlation (Bermudez-Edo et al., 2018).

### 2.6 Modeling

In this study, the entire dataset (360) was randomly divided into a modeling set (300) and a validation set (60) (5: 1) to ensure the generality and stability of the model (Joseph, 2022). The results of statistical analysis of the entire dataset, the modeling set, and the validation set (**Table 2**) showed that the modeling set had a larger data range than the validation set and that the mean, minimum, maximum, standard deviation, and coefficient of variation of the modeling and validation sets were very similar. This indicates that the division of modeling and validation sets are very uniform, which is conducive to the accurate evaluation of model performance (Wu, 2023).

**Table 2:** Statistics of the modeling and validation sets.

Data set	Sample size	Maximum	Minimum	Mean	Standard deviation	Coefficient of variation
Total set	360	6.36	0.505	3.48	1.54	44.3
Modeling set	300	6.36	0.519	3.69	1.56	46.2
Validation set	60	6.16	0.505	3.37	1.49	40.5

**Figure 2:**
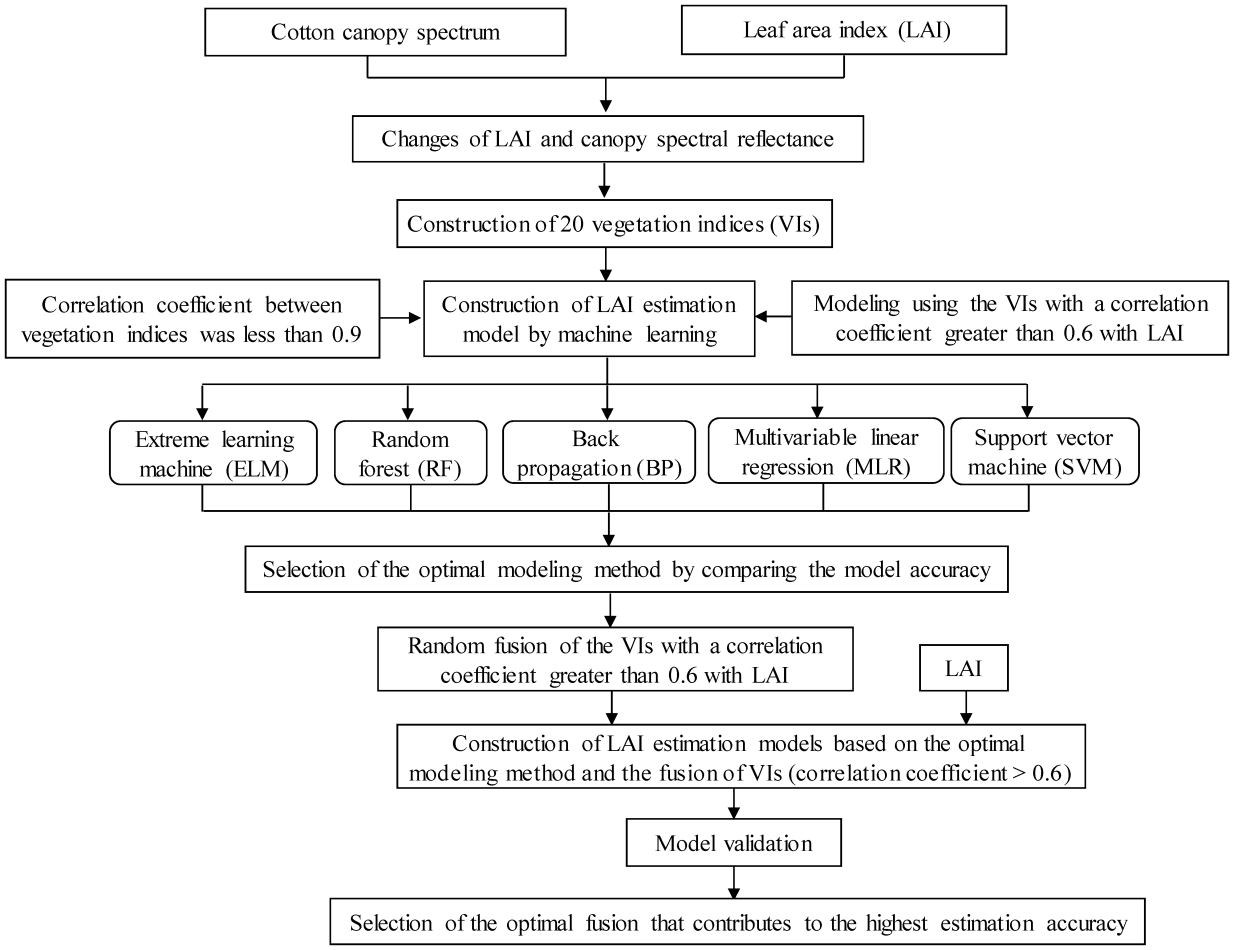
Flowchart of modeling.

Multiple VIs with high correlation with LAI were selected to construct ELM, RF, BP, MLR, and SVM models for LAI estimation using the Scikit-Learn package in Python. Then, the accuracy of the constructed models were compared, and the cross-validation was performed to select the optimal modeling method. Based on the optimal model, the 20 VIs (**Table 1**) were randomly arranged and fused, and the optimal fusion was selected. **Figure 2** shows the specific workflow. In this study, the change trend of the spectral reflectance of cotton canopy and LAI during the whole growth period were first analyzed, and then, 20 VIs related to LAI were selected based on previous studies. After that, the correlations between LAI and VIs were analyzed, and the VIs with a correlation coefficient with LAI of no less than 0.6 (Meng et al., 2021) and a correlation coefficient with other VIs of less than 0.9 were selected (Pasternak and Pawluszek-Filipiak, 2022). Then, the LAI estimation models based on machine learning (ELM, RF, BP, MLR, and SVM) were constructed and compared to select the optimal model. Finally, the VIs participating in the modeling were randomly arranged and fused (i.e., for index 1, 2, and 3, there were seven fusions including 1, 2, 3, 1 + 2, 1 + 3, 2 + 3, and 1 + 2 + 3), and the model was verified to select the best vegetation index fusion model.

#### 2.6.1 ELM model

ELM is an algorithm based on the SLFN. It is characterized by random generation of connection weights between input and hidden layers and thresholds of hidden layer neurons. No special operation is required in training, and the only value is the hidden layer neuron number. The global optimal solution is obtained after completing training (Sun and Gao, 2022).

Given N samples (*x_i_*, *y_i_*) (*_i_* ≤ N, $x_{i}\in R^{n}$ and $x_{i}\in R^{m}$ ), *x_i_* is the input, *y_i_* is the output (expected value). Then, an SLFN with *L* hidden layer nodes can be obtained by following Equation (2):


2 $$\sum_{i=1}^{L}\beta_{i}\;f(\omega_{i}\cdot x_{j}+b_{j})=O_{j}\;1\leq j\leq N$$

where $\beta_{i}={\left(\beta_{i1},\beta_{i2},\cdots ,\beta_{im}\right)}^{T}$ is the output weight matrix for hidden nodes and output nodes, *f(x)* is the activation function, *w_i_* and *x_j_* are inner products, and *o_j_* is the value of the output.

#### 2.6.2 RF model

RF is a learning algorithm integrating multiple CART decision trees. The decision tree model is constructed by randomly selecting multiple samples from the raw samples by bootstrap resampling. There are two key parameters involved in the RF modeling: mtry and ntree. mtry is the number of node splits each time the decision tree model is reconstructed. ntree is the number of decision trees. Through voting on the output of each decision tree, classification can be achieved (Liu et al., 2021b). In regression prediction, the predicted value is the average of the outputs of all trees as shown in Equation (3).


3 $$h\left(x\right)=\frac{1}{k}\sum_{i=1}^{k}h\left(x,\;\theta_{i}\right)$$

where *h(x)* is the predicted value, *θ_i_* is an independently distributed random vector that determines the growth of the decision tree, *x* is the input matrix, *h(x, θ_i_)* is the output of the *i*th regression tree, and *k* is the number of regression trees.

#### 2.6.3 BP neural network model

BP neural network has a good performance in nonlinear fitting and is widely used in classification and regression. In BP neural network modeling, prior assumption is not required. The ReLu function is used as the activation function by the hidden layer, and the number is 1. The linear function and Adam are used as the activation function and the optimizer, respectively, by the output layer. The node number is determined according to Jiang et al. (2021), and the number of nodes of the output layer and hidden layer is determined by an iterative loop. In this study, the iterations were 100, 500, and 1,000. In addition, the learning rates were 0.01, 0.001, and 0.0001.

#### 2.6.4 SVM model

SVM has been widely used in crop inversion research for its high accuracy and generalization ability for small sample data (Hosseini et al., 2021). It can map the nonlinear separable data to the kernel function-created high-dimensional feature space, and construct a linear classification equivalent to a nonlinear classification in the input space. In this study, kernel function, kernel coefficient (γ), and regularized coefficient (C) were {poly, RBF, sigmoid}, 10^−8^–10^8^, and 10^−8^–10^8^, respectively.

#### 2.6.5 MLR model

MLR predicts dependent variables through regression by the optimal combination of multiple independent variables. Multiple linear fitting can predict the relationship between multiple types of known independent variables and their corresponding single dependent variables (Meerasri and Sothornvit, 2022). The expression of MLR is as follows (Equation 4):


4 $$y=w_{0}x_{0}+w_{1}x_{1}+\ldots +w_{n}x_{n}$$

It can be shortened to the following matrix (Equation 5):


5 y=xw

where *y* is the dependent variable (LAI), *x* is the independent variable (vegetation indices), and *w* is the coefficient of the independent variable.

### 2.7 Model validation

In this study, the R^2^, RMSE, nRMSE, and MAE were used to assess the model accuracy. The larger the R^2^, the better the model fit; the smaller the RMSE, nRMSE, and MAE, the higher the model accuracy. The calculation of R^2^, RMSE, nRMSE, and MAE was based on the research of Wang et al. (2022) and Jiang et al. (2023).

## 3 Result

### 3.1 Variation of cotton LAI at different growth stages

**Figure 3:**
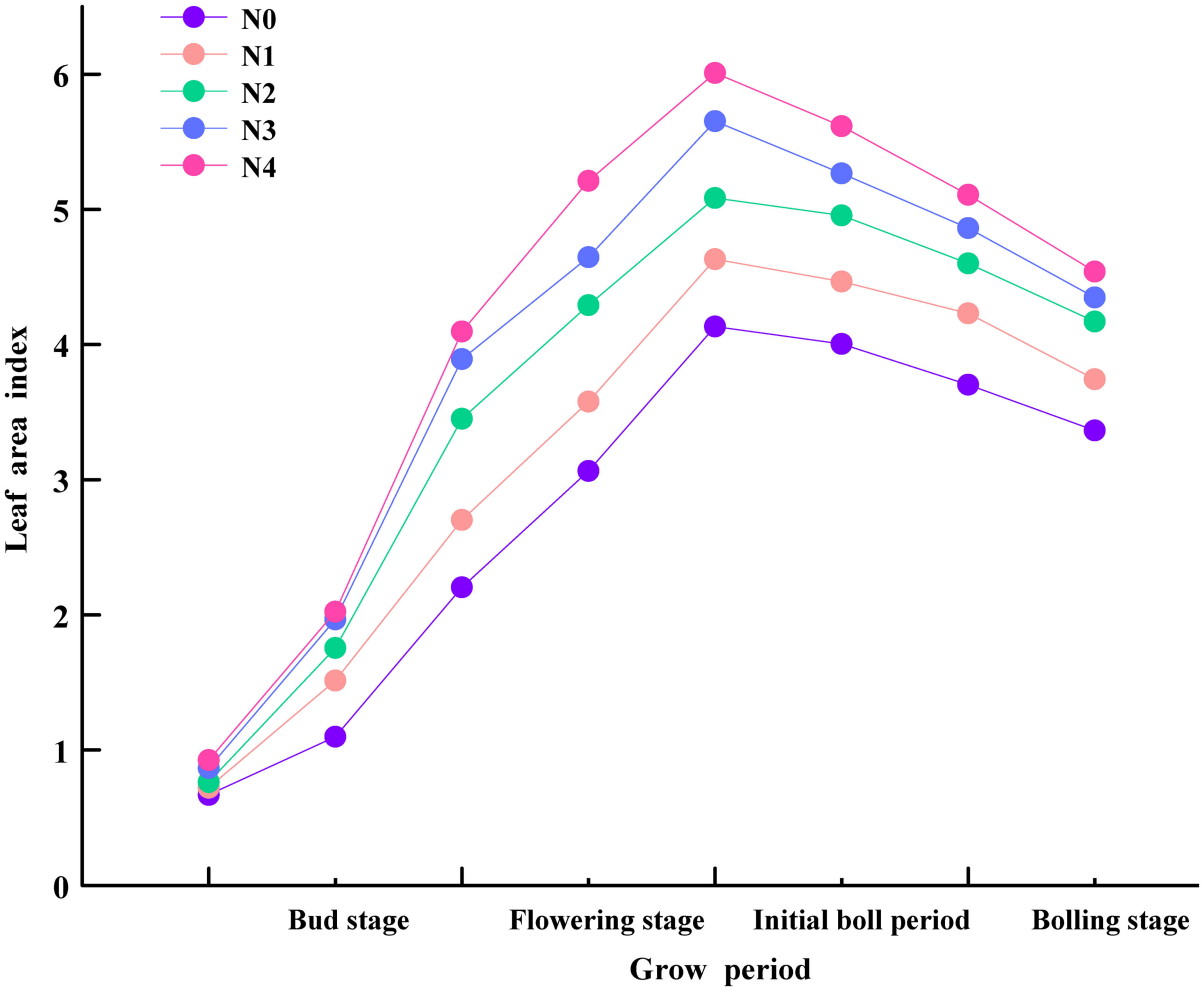
Cotton leaf area index (LAI) variations under N treatments.

Cotton LAI increased first and then reduced (**Figure 3**), and the influence of different N treatments on cotton LAI was great throughout the entire growth period. The more N was applied, the greater was the LAI. The dynamics of cotton LAI in different treatments were similar throughout the entire growth period. The changes of LAI in different treatments were small at the beginning. Then, the LAI increased, and the difference in LAI between different treatments also increased. The LAI peaked in the late flowering stage and then declined.

### 3.2 Changes in cotton canopy spectra

**Figure 4:**
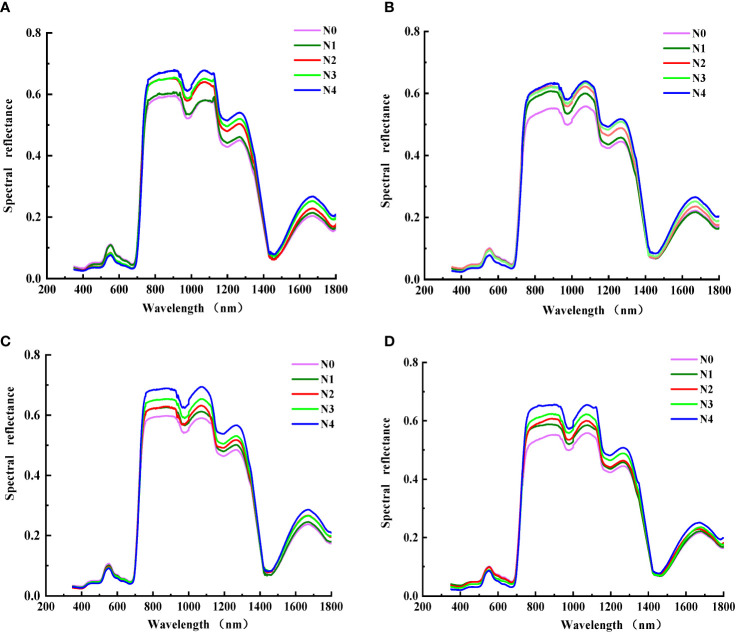
Cotton canopy spectral reflectance under N treatments [**(A)** budding stage; **(B)** flowering stage; **(C)** initial bolling stage; (**D)** full bolling stage].

The spectral reflectance changed consistently in different treatments (**Figure 4**). In the visible region, there was little difference. With the growth of N dose, the spectral reflectance declined (N0 > N1 > N2 > N3 > N4). In addition, the reflectance varied obviously among stages in the NIR region.

In the bud stage, the reflectance of the N0 and N1 treatments were low (N0< N1) and that of the N2, N3, and N4 treatments were high (N4 > N3 > N2) in the NIR region. In addition, there was a significant difference in the reflectance between different treatments throughout the entire growth period. The reflectance increased with the increase in N dose (N4 > N3 > N2 > N1 > N0).

### 3.3 Correlation analysis between cotton LAI and vegetation indices

**Figure 5:**
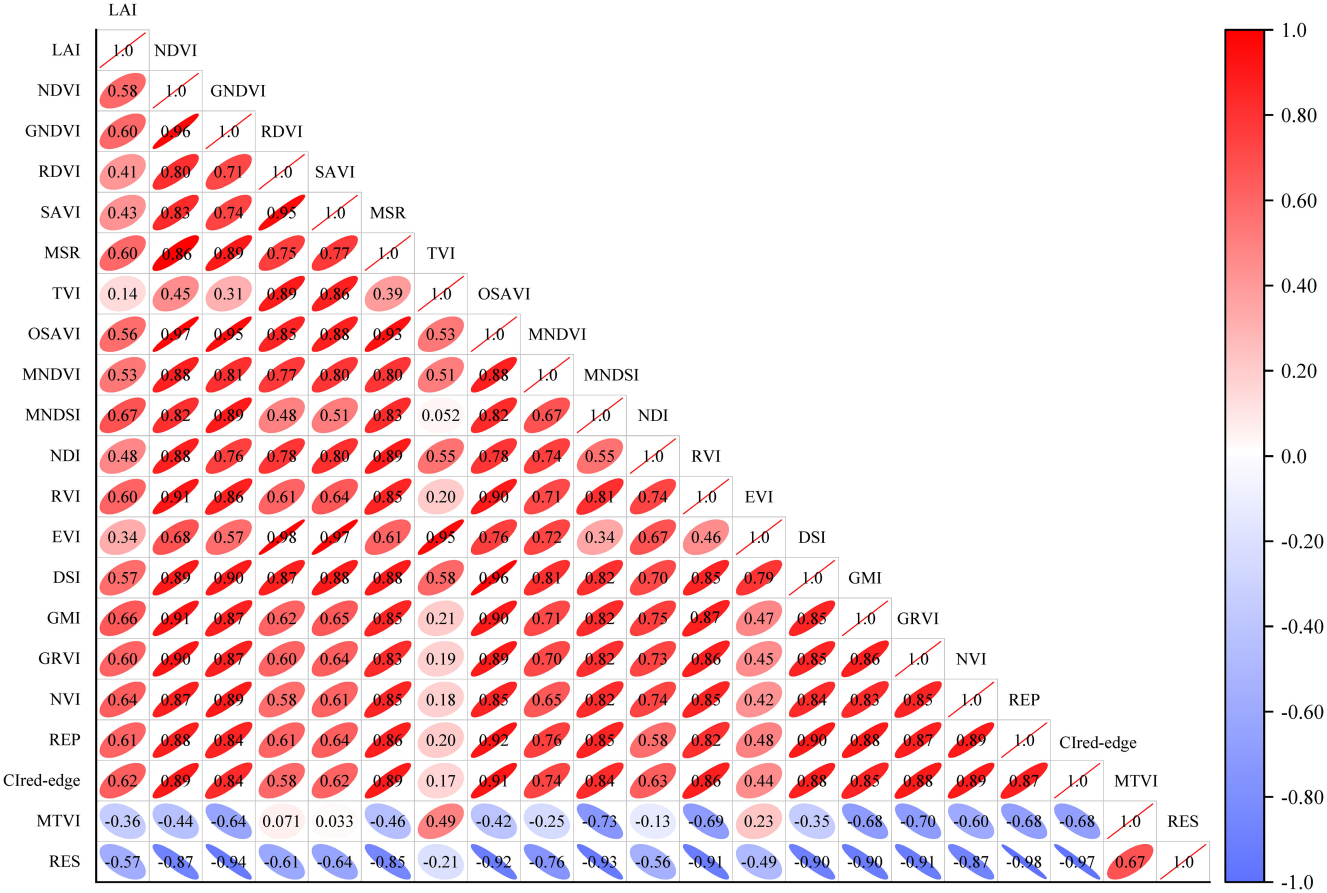
Correlation analysis between vegetation indices and LAI. The ellipse tilted to the left and colored red represents a positive correlation, and the ellipse tilted to the right and colored blue represents a negative correlation.

The coefficients of correlation between VIs and LAI were 0.14–0.67 (**Figure 5**). LAI had a negative correlation with MTVI and RES and a positive correlation with other VIs. Among them, MNDSI had the highest correlation with LAI (0.67), and TVI had the lowest correlation with LAI (0.14). The correlation between VIs varied greatly. The correlation between SAVI and MTVI was the lowest (0.033), while that between EVI and RDVI and between RES and REP were the highest (0.98). In addition, RES had a positive correlation with MTVI, but a negative correlation with other VIs. MTVI had a positive correlation with RDVI, SAVI, TVI, and EVA, and a negative correlation with other VIs.

### 3.4 Cotton LAI estimation by ML model

The 20 VIs were used to construct cotton LAI prediction models based on a single VI (**Table 3**), and it was found that the accuracy of the models were generally low. To further explore the advantages of multi-VIs fusion and the performance of the constructed models, considering the multicollinearity between VIs and the overfitting of the model, this study selected the VIs with a correlation coefficient with LAI of not less than 0.6 and a correlation coefficient with other VIs of less than 0.9 (results of *Section 3.3*) for modeling (**Table 4**), including: GNDVI, MSR, MNDSI, RVI, SRI, GRVI, NVI, REP, and CIred-edge. The comparison of the ELM, RF, BP, LR, and SVM modeling showed that the RF models had the highest accuracy, with R^2^, MAE, RMSE, and NRMSE being 0.87, 0.37, 0.57, and 0.16, respectively, followed by the ELM, BP, and LR models. SVM models had the lowest accuracy, with R^2^, MAE, RMSE, and NRMSE being 0.51, 0.84, 1.11, and 0.32, respectively.

**Table 3:** Accuracy of cotton LAI estimation models constructed based on different modeling methods and 20 VIs.

Model	Variables	R^2^	MAE	RMSE	NRMSE
ELM	NDVI, GNDVI, RDVI, SAVI, MSR, TVI, OSAVI, MNDVI, MNDSI, NDI, RVI, EVI, DSI, SRI, GRVI, NVI, REP, CIred-edge, MTVI, RES	0.37	1.09	1.32	0.38
RF		0.39	1.07	1.29	0.37
BP		0.35	1.10	1.34	0.38
MLR		0.34	1.17	1.38	0.40
SVR		0.32	1.19	1.41	0.41

**Table 4:** Accuracy of cotton LAI estimation models constructed based on the vegetation indices with a correlation coefficient of not less than 0.6 with leaf area index.

Model	Parameters	Variables	R^2^	MAE	RMSE	NRMSE
ELM	neurons(40, tanh)	GNDVI, MSR, MNDSI, RVI SRI, GRVI, NVI, REP, CIred-edge	0.71	0.63	0.90	0.26
RF	(n_estimators:14, max_depth:63)		0.87	0.37	0.57	0.16
BP	hidden_layer_sizes:10;solver:lbfgs;		0.61	0.70	1.02	0.29
	random_state:0; max_iter:500;					
MLR	–		0.54	0.83	1.09	0.32
SVM	Kernel: rbf; Cost:0.3;gamma:30;		0.51	0.84	1.11	0.32

### 3.5 Construction of cotton LAI estimation models based on RF and multi-vegetation indices fusion

The selected nine VIs were randomly arranged and fused. When there were 1, 2, 3, 4, 5, 6, 7, 8, and 9 VIs for fusion, 1, 15, 15, 15, 15, 15, 15, 9, and 1 combinations were randomly selected, respectively. Only the optimal fusions under different number of VIs are shown here. It was found that the RF model had the highest accuracy when MNDSI, SRI, GRVI, REP, CIred-edge, MSR, and NVI were fused (**Table 5**). The RF-7 model had the highest R^2^ (0.90) and the smallest RMSE (0.50), NRMSE (0.14), and MAE (0.26). The accuracy of the models built by a single VI was the lowest, with R^2^, MAE, RMSE, and NRMSE being 0.63, 0.82, 1.03, and 0.30, respectively.

**Table 5:** Accuracy of the optimal RF models for cotton LAI estimation when different vegetation indices are fused.

Model	Vegetation indices	R^2^	MAE	RMSE	NRMSE
RF-9	GNDVI, MNDSI, SRI, GRVI, REP, CIred-edge, MSR, RVI, NVI	0.87	0.41	0.56	0.16
RF-8	GNDVI, MNDSI, SRI, GRVI, REP, CIred-edge, MSR, RVI	0.89	0.33	0.52	0.15
RF-7	MNDSI, SRI, GRVI, REP, CIred-edge, MSR, NVI	0.90	0.26	0.50	0.14
RF-6	GNDVI, MNDSI, SRI, GRVI, REP, RVI	0.87	0.34	0.57	0.16
RF-5	MNDSI, SRI, REP, MSR, NVI	0.74	0.63	0.84	0.24
RF-4	MNDSI, GRVI, CIred-edge, NVI	0.71	0.72	0.96	0.28
RF-3	CIred-edge, RVI, NVI	0.69	0.74	0.96	0.27
RF-2	MNDSI, NVI	0.68	0.74	0.93	0.27
RF-1	MNDSI	0.63	0.82	1.03	0.30

### 3.6 Model validation

**Figure 6:**
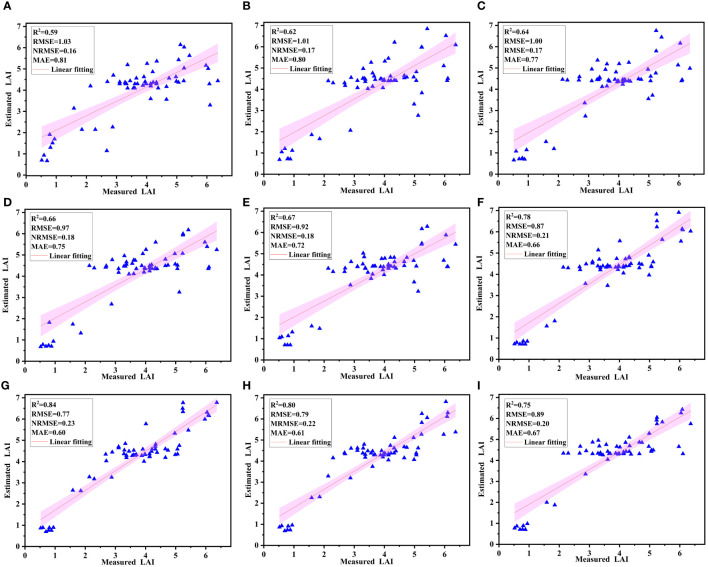
Fitting results of the measured and the RF model predicted LAI based on validation set **(A–I)** represent the optimal fusions when one to nine VIs were fused).

The results of linear fitting of predicted LAI by RF models (**Table 5**) and measured LAI (**Figure 6**) showed that the slope of the fitted line of measured LAI and RF-7 was closest to 1, and the slope of the fitted lines of measured LAI and single VIs was the lowest (**Figure 6A**). When 1, 2, 3, 4, 5, 6, and 7 VIs were fused (**Figures 6A–G**), the model accuracy increased with the increase in the number of VIs fused, but the accuracy decreased when 8 and 9 VIs were fused (**Figures 6H, I**). The slopes of all curves were less than 1, indicating that there was no over-fitting.

## 4 Discussion

### 4.1 Variation of cotton LAI and canopy spectrum under different nitrogen treatments

Leaf area index reflects the energy flow and material cycle in plant canopy and is closely related to crop yield. Therefore, it is commonly used in the study of photosynthesis, respiration, and carbon accumulation of vegetation (Lu et al., 2020; Sun et al., 2023). In this study, the cotton LAI under different N treatments showed an unimodal variation trend. This reflects that N has an obvious regulatory effect on cotton LAI, which is related to the growth characteristics of cotton at different stages (Ninkuu et al., 2023). From bud to flowering stage, due to the increase in cotton branches and leaves, the cotton leaf area continues to increase, resulting in a rapid increase in LAI, and the difference in LAI between N treatments also increases. This is similar to the results of Wen (2016). Nitrogen is the main nutrient required for cotton growth, and sufficient N supply could promote leaf enlargement. The bud stage is a key period for cotton vegetative growth. With the increase in temperature, cotton growth accelerates. This ultimately leads to increased LAI. During the flowering stage, cotton enters into the reproductive growth stage, and a large amount of organic matter is transferred to reproductive organs. In the late flowering stage, LAI is the largest, and then, LAI gradually declines in the bolling stage. However, Kumar et al. (2022) found that LAI began to decrease after the boll-opening stage. On the one hand, it may be caused by the different temperature, sunshine time, and rainfall in different regions (high temperature and long sunshine time can accelerate cotton growth, but rainfall slows down cotton growth). On the other hand, the increasing competition between vegetative and reproductive growth in the flowering and bud stages leads to leaf shedding and decreased LAI (Li, 2022). Especially, in this study, the LAI declined fastest in the N4 treatment. High N dose leads to too vigorous vegetative growth. The lower leaves block each other, leading to poor ventilation and early leaf aging and shedding. In addition, in our previous report (Fan et al., 2023), the LAI of cotton changed most significantly under the N3 treatment, while the LAI changed the most under N4 treatment in this study. This may be due to the fact that the N4 in this study was 440 kg N ha^−1^. However, in previous study, the N3 was 360 kg N ha^−1^ and the N4 was 480 kg N ha^−1^. Although the N application rate N4 is the largest in this study, it is lower than that in previous study. This indicates that the demand for N fertilizer in cotton is limited, and it is not that the more nitrogen is fertilized, the greater the LAI.

The uptake, transport, and assimilation of N show dynamic changes in cotton leaves, and the nutrient composition and content in leaves also change continuously, resulting in changes in leaf color, morphology, and spectral reflectance (Sun et al., 2022). In the visible region, there was no significant difference in canopy spectral reflectance at different stages, and the reflectance decreased with the growth of N dose. In the NIR region, the difference increased, and the reflectance increased with the growth of N dose. This is consistent with the results of Liu et al. (2019). However, in our previous study (Fan et al., 2023), the canopy spectral reflectance reached the maximum under N3 treatment. In this study, the canopy spectral reflectance was the largest under N4 treatment. This may be due to the fact that although the N4 in this study is the largest, it is still lower than the N4 in the previous study. Excessive or small nitrogen fertilizer application can lead to a decrease in LAI, thereby reducing the canopy spectral reflectance.

The spectral reflectance of visible region mainly reflects leaf pigment status. In crop photosynthesis, chlorophyll reflects green light and absorbs red and violet light, resulting in low canopy spectral reflectance (Li et al., 2019; Yang et al., 2022). The reflectance in the NIR region is mainly affected by optical properties and leaf structure. In addition, the differences in intercellular space, composition, and shape are also influencing factors (Richetti et al., 2019; Spafford et al., 2021). Thus, the reflectance of the NIR region gradually reduces over time.

### 4.2 Construction of cotton LAI estimation model based on the fusion of multiple vegetation indices

Vegetation index can provide crop growth information. At present, a large number of remote sensing studies of cotton LAI based on a single vegetation index have been carried out (Yan et al., 2022). In this study, the correlation analysis of LAI and VIs was carried out. It was found that MNDSI had the highest correlation with LAI. This is consistent with the results of Gao et al. (2013). MNDSI can better reflect the status of cotton LAI. The spectral bands in MNDSI has a strong absorption on the spectra of cotton LAI (Li et al., 2018). Therefore, future studies can try to accurately predict cotton LAI changes through this index. In this study, the correlations between some VIs were very high, with a correlation coefficient of 0.98 (EVI and RDVI, RES and REP). This may be due to the fact that these VIs contain the same spectral bands, resulting in very high multicollinearity between the VIs, so it is necessary to filter the VIs before fusion.

It was found that the ML models based on 20 VIs had a low accuracy. This may be due to the high correlations between some VIs. Therefore, the VIs with a correlation coefficient not less than 0.6 were selected for modeling. It was found that this improved the prediction accuracy of all models (ELM, RF, BP, MLR, and SVM). This indicates that LAI has its unique spectral features and corresponding VIs. Among the models, the RF models had the highest accuracy. This is consistent with the results of Han et al. (2019) and Wang et al. (2021). RF is a tree-based ensemble learning. It higher accuracy in this study may be due to that the data are trained using randomly selected subsets at each node, and the best performing prediction variables are selected to split nodes (insensitive to noise), which can effectively solve problems such as overfitting and collinearity (Lu and He, 2019).

In this study, multiple VIs were randomly arranged and fused, and the optimal fusion was selected for RF modeling. It was found that the model accuracy constructed using VI fusion was higher than that of the model constructed using a single VI. This is similar to the results of Qi et al. (2020) and Rumora et al. (2021). This may be due to that each VI is a physical structure and has limited valid bands (only two to three bands). It was also found that the model accuracy based on the fusion of seven VIs (RF-7) had the highest accuracy. This indicates that the fusion of the seven VIs synthesizes the structural characteristics of each VI and contains more reflectance information, which ultimately improves the LAI prediction accuracy. However, when eight and nine VIs were fused, the model accuracy began to decrease. This may be due to that (1) different VIs have different sensitivities to the same parameter; the model accuracy increases when the highly sensitive VIs are fused for modeling, and vice versa. (2) The influences of canopy structure, cover, and soil background brightness on different VIs are also different (Qiao et al., 2022). (3) There is data redundancy between VIs, which affects the model accuracy. Therefore, appropriate VI fusion can eliminate the influence of environmental factors, realize the complementarity of spectral features, and increase vegetation information, which ultimately improves model accuracy and stability.

In addition, hyperspectral data are more informative than VIs. It is widely used in different remote sensing analysis and feature classification tasks because hyperspectral data covers a wide range of bands and contains all the spectral information of features. However, it faces the problem of information redundancy, which increases the complexity of data processing and analysis, and may reduce the interpretability of the model (Jiang et al., 2022). In addition, sensitive wavelengths are more susceptible to interference from non-target factors, such as soil background and light conditions (Meiyan et al., 2023). In contrast, the VI is usually based on the combination of specific wavelengths, which can effectively eliminate the interference of soil signals and other factors, and is specially used for the monitoring of vegetation parameters. It is well targeted and has a high interpretability for vegetation-related research (Cao et al., 2021).

At present, the vegetation canopy spectral acquisition instruments mainly include hyperspectral sensors and multispectral (RGB) sensors. The wide spectral range of hyperspectra, including visible light and near-infrared spectra, provides rich spectral information, which enables hyperspectral data to capture detailed spectral characteristics of features (Liu et al., 2023a), such as spectral reflectance, absorption peaks, and absorption valleys. These detailed spectral features make hyperspectral data have higher performance and application potential in object detection. However, the number of RGB bands is small and contains less information (Liu et al., 2022a), making it difficult to fully reflect the changes in the spectral features of vegetation parameters. Nevertheless, due to the processing complexity and high cost of hyperspectral data, RGB still has certain advantages in some scenarios, especially in vegetation monitoring and simple object classification tasks. For example, Liu et al. (2022b) improved the estimation accuracy of potato aboveground biomass (AGB) by extracting different texture features from RGB images.

In recent years, many scholars have preprocessed hyperspectral data and achieved good estimation results. For example, Liu et al. (2022c) used the Savitzky-Golay (SG) smoothing to smooth the spectrum collected by unmanned aerial vehicles (UAVs) and the random forest to extract the characteristic bands, and found that the PLSR model had the highest estimation accuracy for AGB. Liu et al. (2023b) extracted spectral features from UAV-collected RGB images and hyperspectral data through wavelet transform and found that the fusion of the features of RGB images and hyperspectral data could improve the estimation accuracy of AGB. Since hyperspectra can provide comprehensive spectral information, future research can transform the raw hyperspectral data to highlight spectral features, extract features by some methods to reduce data redundancy, and calculate the vegetation index based on the spectral features to construct a model. In addition, RGB images and hyperspectral data can be combined to explore the potential of multi-source remote sensing data fusion in predicting cotton LAI.

## 5 Conclusions

In this study, the variations in cotton canopy spectral reflectance and LAI were analyzed, and the ML models for cotton LAI prediction were constructed by multi-VI fusion, to explore the optimal VI fusion that contributes to a high cotton LAI prediction accuracy. Cotton LAI raised first and then reduced throughout the entire growth period under different N treatments. In the visible region, no obvious difference in reflectance was detected among growth stages, and the spectral reflectance decreased with the growth of nitrogen dose. However, it was opposite in the NIR region. The correlations between different VIs and LAI are different, and the correlations between some VIs are very high, resulting in data redundancy. The RF model constructed based on multi-VI fusion had the highest accuracy, while the accuracy of the SVM model was the lowest. In addition, the RF model constructed based on the fusion of MNDSI, SRI, GRVI, REP, CIred-edge, MSR, and NVI had the highest accuracy, with R^2^, MAE, RMSE, and NRMSE of 0.90, 0.26, 50, and 0.14, respectively. The validation results of the optimal model showed that the R^2^, MAE, RSME, and NRMSE were 0.84, 0.60, 0.77, and 0.21, respectively. Therefore, fusion of appropriate VIs can increase the number of spectral features and further improve the cotton LAI prediction accuracy. However, the number of VIs constructed in this study is limited, and the MLs chosen are traditional. Therefore, more VIs will be constructed in the future, and more deep learning methods will be applied to optimize the VI fusion and the LAI prediction model.

## Data availability statement

The original contributions presented in the study are included in the article/**Supplementary Material**. Further inquiries can be directed to the corresponding authors.

## Author contributions

XF: Conceptualization, Data curation, Formal analysis, Investigation, Methodology, Visualization, Writing – original draft. MZ: Software, Writing – review & editing. HC: Validation, Writing – review & editing. LZ: Supervision, Writing – review & editing. ZZ: Supervision, Writing – review & editing. JW: Investigation, Writing – review & editing. XL: Conceptualization, Funding acquisition, Methodology, Resources, Supervision, Writing – review & editing. PG: Conceptualization, Funding acquisition, Methodology, Resources, Supervision, Writing – review & editing. QZ: Methodology, Funding acquisition, Writing – review & editing. LM: Investigation, Funding acquisition, Writing – review & editing.
