# Supplementary figures and images for: The fusion of vegetation indices increases the accuracy of cotton leaf area prediction
Source: Front Plant Sci. 2024 Jul 4;15:1357193. doi: 10.3389/fpls.2024.1357193 (PMC11298913; doi:10.3389/fpls.2024.1357193)

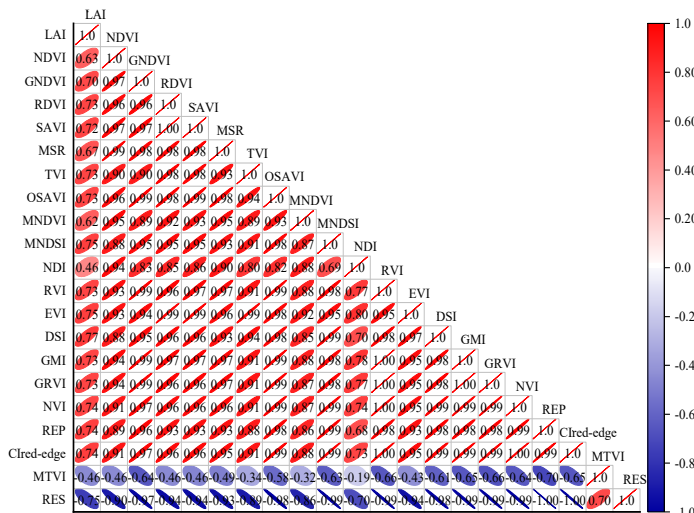

Bud stage

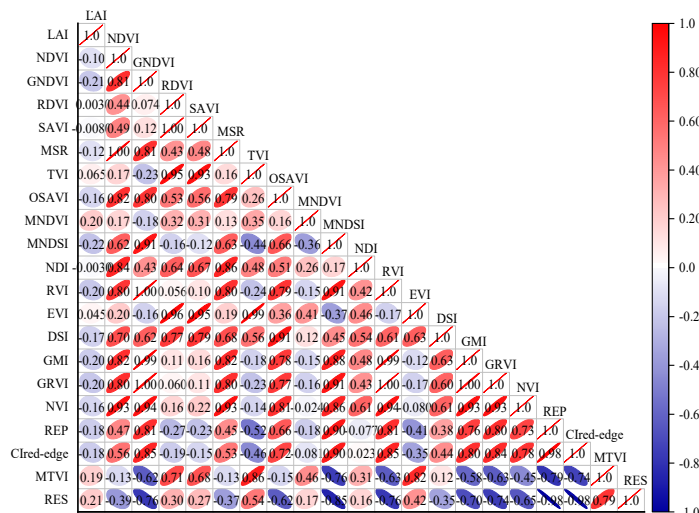

Flowering stage

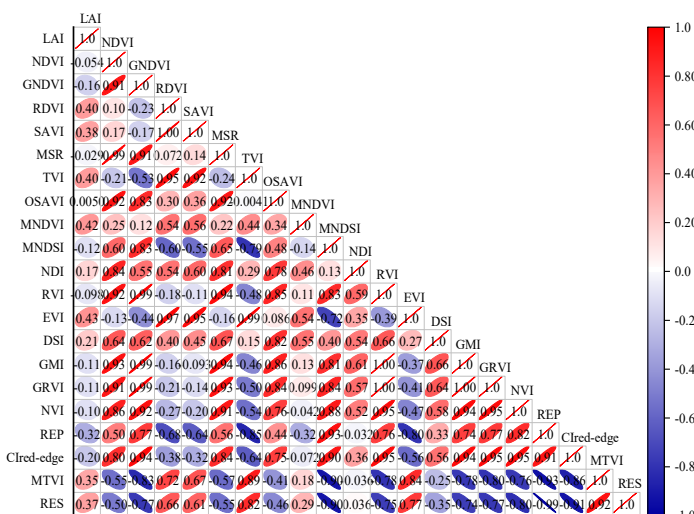

Initial boll period

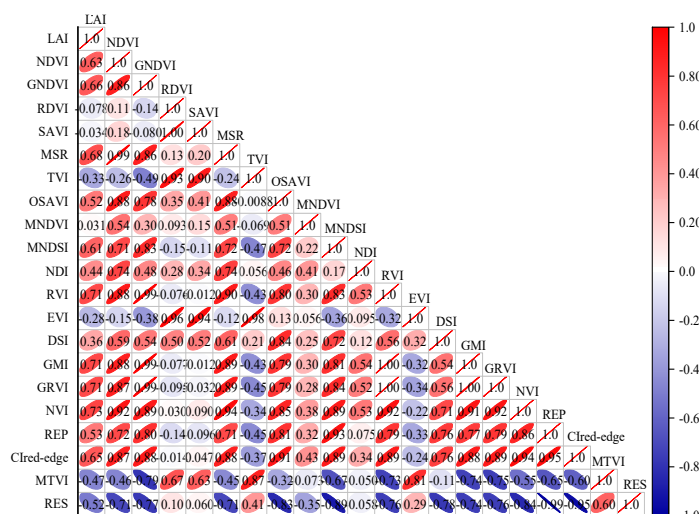

Supplement: Supplementary file 1 [file DataSheet_1.pdf]
